# Average absorbed breast dose (2ABD): an easy radiation dose index for digital breast tomosynthesis

**DOI:** 10.1186/s41747-020-00165-2

**Published:** 2020-07-07

**Authors:** Antonio C. Traino, Patrizio Barca, Rocco Lamastra, Raffaele M. Tucciariello, Chiara Sottocornola, Carolina Marini, Giacomo Aringhieri, Davide Caramella, Maria E. Fantacci

**Affiliations:** 1 U.O.Fisica Sanitaria, Azienda Ospedaliero-Universitaria Pisana, Via Roma n.67, 56125 Pisa, Italy; 2 Dipartimento di Fisica E.Fermi, Università di Pisa, L.go B.Pontecorvo n.3, 56127 Pisa, Italy; 3 U.O.S.D. Fisica Sanitaria, Azienda Usl Toscana Sud-Est, Ospedale San Donato, Via P. Nenni 20, 52100 Arezzo, Italy; 4 S.D.Radiologia Senologica, Azienda Ospedaliero-Universitaria Pisana, Via Roma n.67, 56125 Pisa, Italy; 5 Radiologia Diagnostica e Interventistica, Università di Pisa, Via Paradisa n.2, 56100 Pisa, Italy

**Keywords:** Digital breast tomosynthesis, Mammography, Phantoms (imaging), X-rays

## Abstract

**Background:**

To propose a practical and simple method to individually evaluate the average absorbed dose for digital breast tomosynthesis.

**Methods:**

The method is based on the estimate of incident air kerma (*k*_*a,i*_) on the breast surface. An analytical model was developed to calculate the *k*_*a,i*_ from the tube voltage, tube load, breast thickness, x-ray tube yield, and anode-filter combination. A homogeneous phantom was employed to simulate the breast in experimental measurements and to assess the dose-depth relationship. The *k*_*a,i*_ values were employed to calculate the “average absorbed breast dose” (2ABD) index. Four mammographic units were used to develop and test our method under many conditions close to clinical settings. The average glandular dose (AGD) calculated following the method described by Dance et al., and the 2ABD computed through our method (*i.e.,* from the exposure parameters) were compared in a number of conditions.

**Results:**

A good agreement was obtained between the *k*_*a,i*_ computed through our model and that measured under different clinical conditions: discrepancies < 6% were found in all conditions. 2ABD matches with a good accuracy the AGD for a 100% glandular-breast: the minimum, maximum, and mean differences were < 0.1%, 7%, and 2.4%, respectively; the discrepancies increase with decreasing breast glandularity.

**Conclusions:**

The proposed model, based on only few exposure parameters, represents a simple way to individually calculate an index, 2ABD, which can be interpreted as the average absorbed dose in a homogeneous phantom, approximating a 100% glandular breast. The method could be easily implemented in any mammographic device performing DBT.

## Key points

- A dosimetry index (average absorbed breast dose, 2ABD) for digital breast tomosynthesis (DBT) is presented.
- 2ABD is a physical quantity directly computable from measurable quantities.
- 2ABD could be easily implemented on clinical mammographic devices performing DBT.

## Background

Digital mammography (DM) represents the reference x-ray imaging technique for the early detection and diagnosis of breast cancer. It is a fast and low radiation dose modality, which allows to explore the breast with a diagnostic performance enabling to reduce breast cancer mortality [1].

The main limitation of DM is its intrinsic two-dimensional nature, resulting in tissue overlapping which can lead to limited sensitivity and specificity. In order to reduce these limitations and improve accuracy, two new x-ray-based imaging modalities have been developed: dedicated breast computed tomography [2] and digital breast tomosynthesis (DBT) [3, 4]. To date, however, only DBT has been introduced in clinical routine worldwide: different DBT systems have received approval for clinical use around the world and their employment increased in recent years [4–6].

DBT produces pseudo-three dimensional images by acquiring a limited number of projections of the breast from a limited angular range. Similarly to DM, the breast is compressed and held stationary between the compression paddle and the detector. The x-ray tube rotates in one plane around the compressed breast, over a limited angular range, and a projection every few degrees is acquired [4]. Depending on the specific DBT device, a filtered back-projection or an iterative reconstruction algorithm is applied to the acquired projections, and a set of reconstructed slices is produced [5]. Thus, differently from DM, DBT provides tomographic slices of an entire tissue volume, likewise CT scans, and the total number of reconstructed images depends on the thickness of the examined compressed breast.

In DBT, many parameters may influence the breast absorbed dose. Exposure parameters such as kVp and mAs values play the same role in DBT as in DM; however, the number of projections and the x-ray tube angular range are main factors to be considered for DBT only [7].

The current reference dosimetry index employed to estimate the radiation dose in DM is the average glandular dose (AGD), which is representative of the absorbed dose by the glandular tissue, which is more radiosensitive than skin and adipose tissue [8, 9]. In DBT, the concept of AGD has been extended to take into account each projection at a given angle [10–12]. More in detail, a “tomo” factor T_f_ was introduced to incorporate the angular aspects of DBT data acquisition in the method proposed by Dance et al. [11]. The T_f_ factor has been provided as a function of breast thickness, ranging from 2 to 11 cm at 1-cm step [11]. The AGD method requires measurements of incident air kerma (*k*_*a,i*_) and a number of correction and conversion factors must be applied to *k*_*a,i*_ to obtain the AGD [11]. These factors are calculated from Monte Carlo simulations, but they cannot be directly measured.

In a previous study, a practical and simple approach based on measurable physical quantities was introduced to evaluate a quantity called average absorbed breast dose (2ABD) in DM [13]. The aim of this work was to extend the 2ABD method to DBT and to perform a dosimetry comparison between 2ABD and AGD evaluated by the Dance method.

## Methods

### Extension of the 2ABD model to DBT

To evaluate 2ABD for DBT, we followed the same approach developed for DM procedures [13].

Four mammographic devices were utilised: two Selenia Dimensions (Hologic, Bedford, Mass, USA), devices A and B and two Amulet Innovality (Fujifilm Medical System Inc., USA), devices C and D. Device A was used to develop and validate our method through experimental measurements. Therefore, this device was chosen as reference. Additionally, in order to further test our method, a set of measurements was performed on the other three devices.

The Selenia Dimensions model performs both DM and DBT and offers three anode-filter combinations (W-Rh or W-Ag for DM, W-Al for DBT). The DBT angular range of the x-ray tube is ± 7.5°. The Amulet Innovality model employs the W-Rh anode-filter combination for DM and the W-Al anode-filter combination for DBT; two acquisition modes can be selected: the standard mode with a ± 7.5° x-ray tube angular range and the high-resolution mode with a ± 20° x-ray tube angular range.

The 2ABD calculation method for DBT was developed starting from two main approximations: a homogeneous phantom (polystyrene, C_8_H_8_, with admixture of 2.1 ± 0.2% of TiO_2_) with planar dimensions of 16 × 16 cm^2^, and variable thickness was employed to simulate the breast in experimental measurements; the beam attenuation was expressed as a function of the phantom depth following the exponential decay model.

Under these assumptions, the 2ABD was defined as follows:

1 $$ 2\mathrm{ABD}\approx \frac{1}{T}\cdot {\int}_0^T{k}_{a,i}\cdot C\cdot \exp \left(-m\cdot x\right)\kern0.5em dx $$

where *T* is the breast thickness, *k*_*a,i*_ is the incident air kerma on the breast/phantom surface, *m* is a parameter related to the beam attenuation in the phantom, and *C* is a conversion factor from the *k*_*a,i*_ to dose in the phantom. The factor *C* accounts also for the backscatter contribution to the *k*_*a,i*_. Specifically, the C factor is given by: $C=B\bullet {\left(\frac{\overline{\mu_{\mathrm{en}}}}{\rho}\right)}_{air}^{ph}$, where *B* is the backscatter factor and ${\left(\frac{\overline{\mu_{\mathrm{en}}}}{\rho}\right)}_{air}^{ph}$ is the ratio between the mass energy absorption coefficient of the phantom and the mass energy absorption coefficient of air, averaged over the x-ray energy spectrum. The *B* factor was evaluated from experimental measurements, and a value of 1.1 was adopted in this work. The *B* value was obtained by performing two air kerma measurements, with and without the phantom, and taking the ratio of the two detector readings respectively. No appreciable variations were observed in the range 2–9 cm of the phantom thickness. A value of ~ 0.7 was adopted for ${\left(\frac{\overline{\mu_{\mathrm{en}}}}{\rho}\right)}_{\mathrm{air}}^{\mathrm{ph}}$ (no significant variations were observed by varying the tube voltage, for all the anode/filter combination employed in our study). The ${\left(\frac{\overline{\mu_{\mathrm{en}}}}{\rho}\right)}_{\mathrm{air}}^{\mathrm{ph}}$ value was computed by considering the weight average value of the ${\left(\frac{\mu_{\mathrm{en}}}{\rho}\right)}_{\mathrm{air}}^{\mathrm{ph}}$ on the x-ray energy spectrum of the mammographic device. The ${\left(\frac{\mu_{\mathrm{en}}}{\rho}\right)}_{\mathrm{air}}^{\mathrm{ph}}$ values were obtained from the National Institute of Standards and Technology website (https://www.nist.gov/pml/x-ray-mass-attenuation-coefficients). The above mentioned quantities (*k*_*a*, *i*_, *C*, *m*) are required in order to calculate 2ABD in any clinical condition. Therefore, a simple model for estimating *k*_*a,i*_ and *m* was developed.

### Evaluation of *k*_*a,i*_

A set of air kerma measurements were performed on the Selenia Dimensions device in DBT modality through a flat 60-cm^3^ ionisation chamber coupled to an electrometer (20X60E chamber model, 2026C Radcal Corporation®, Monrovia, CA, USA) setting different kVp and mAs values. In order to better simulate the clinical settings, the *k*_*a,i*_ was measured by adopting the closest exposure parameters to the automatic exposure control (AEC) conditions. Additionally, the x-ray tube was free to rotate as in clinical examinations.

The *k*_*a,i*_ at the breast surface depends on tube-voltage, tube load, anode-filter combination, breast thickness, and distance between the x-ray source and the upper surface of the breast. The following relationship was employed:

2 $$ {k}_{a,i}=\eta \cdot \left(\alpha \cdot {\mathrm{kVp}}^2+\beta \cdot k\mathrm{Vp}+\gamma \right)\cdot \mathrm{mAs}\cdot {\left(\frac{\mathrm{FSD}}{\mathrm{FSD}-T}\right)}^2 $$

where FSD is the focus-to-support distance (67.5 cm for the Selenia Dimensions model, 65 cm for the Amulet Innovality model), *T* is the breast thickness, *η* is a correction factor which takes into account differences in the x-ray tube yield (air kerma to tube load ratio) of different mammographic devices. It can be defined as:

3 $$ \eta =\frac{Y_{tb}(FSD)}{Y_0\left({FSD}_0\right)}\cdot {\left(\frac{\left({FSD}_0\right)}{(FSD)}\right)}^2 $$

where *Y*_tb_ represents the yield (mGy/mAs) of the x-ray tube used, and *Y*_0_ is the reference tube yield (*i.e.*, the tube yield of the device A). FSD and FSD_0_ are the distances from the x-ray source at which *Y*_tb_ and *Y*_0_ are evaluated (a reference distance of FSD_0_ = 67.5 cm was chosen in our case for the device A). Both *Y*_0_ and *Y*_tb_ must be evaluated at the same tube voltage (32 kVp in our case). *α*, *β*, and γ are fitting parameters derived from the experimental measurements for a fixed anode-filter combination (W-Al in our case). The choice of 32 kVp as reference tube voltage was due to two main reasons: it is one of the most used voltages at our centre, and it lies in the middle of the tube voltage range used in DBT.

The accuracy of our method was evaluated in a number of exposure settings by comparing the air kerma measured through the ionisation chamber and the *k*_*a,i*_ calculated through Eq. (2).

Uncertainties in measured air kerma were referred to the accuracy of the detector, while uncertainties associated to the parameters involved in Eq. (2) were employed to estimate the final uncertainties of the computed air kerma values.

### Evaluation of *m*

The following exponential relationship was employed to derive *m* as a function of kVp:

4 $$ I(d)={I}_0\cdot \exp \left(-m\cdot d\right) $$

To simulate the breast, a homogeneous phantom with a density of 1.04 ± 0.04 g/cm^3^ (mean ± standard deviation), composed of polystyrene (C_8_H_8_) with admixture of 2.1 ± 0.2 % TiO_2_, consisting of many squared plates (16 × 16 cm^2^) was used. The thickness of each plate was 0.5 or 1.0 cm. The ionisation chamber was placed between the phantom plates to measure the beam intensities *I* at different depths *d*.

Measurements were performed on the Selenia Dimensions equipment (device A) in a range of 26–48 kVp and 40 mAs. The inverse square law was adopted to account for the variations in source-to-chamber distance due to the different depth of the phantom.

The *m* dependence from kVp was expressed as:

5 $$ m=\frac{a}{k{\mathrm{Vp}}^b} $$

where *a* and *b* are fitting parameters.

### Calculation of 2ABD

Once *k*_*a,i*_ from Eq. (2) and *m* from Eq. (5) were evaluated, 2ABD was computed from Eq. (1) in a number of clinical settings. Only the tube voltage, the tube load, the breast thickness, the value of the *η* parameter, and the FSD are required to estimate *k*_*a,i*_ and *m* for a given anode-filter combination and, therefore, to calculate 2ABD. The overall uncertainties in 2ABD calculations were estimated by applying the uncertainty propagation formula for *α*, *β*, *γ*, *η*, kVp, mAs, *T*, and FSD.

The 2ABD calculated by applying Eq. (1)—*i.e.*, by employing only the abovementioned input parameters of the model—was compared to AGD for different breast glandularity. AGD was computed by considering a breast of the same phantom thickness with different breast glandularity and adopting the same exposure parameters of the phantom. Uncertainties in 2ABD were obtained by considering a 4% of accuracy in the ionisation chamber (as reported in the Model 2026C, Radiation Monitor Controller Manual) and by propagating uncertainties of all quantities involved in the calculation. The total uncertainty in AGD calculation was considered to be 20% [14].

### Comparison of 2ABD in DM and DBT procedures

The homogeneous phantom was also employed for comparing the 2ABD values for DM and DBT in AEC conditions on device A. A number of automatic exposures for different phantom thicknesses (from 2.5 to 7.5 cm) were executed in both modalities, and the corresponding input parameters required for the 2ABD calculation were recorded. The 2ABD values were computed according to Eq. (1) for both modalities.

The AGD was calculated as the reference dosimetry index by following the approach by Dance et al. [8] for the same AEC conditions of the 2ABD calculation.

## Results

### Evaluation of *k*_*a,i*_

The comparison between measured and calculated *k*_*a,i*_ according to Eq. (2) for device A is presented in Table 1. The two quantities are in good agreement within the uncertainties and the maximum difference among all considered cases was found to be around -5% of the measured value. The values of the coefficients employed to compute the incident air kerma by Eq. (2) are presented in Table 2.

**Table 1:** Comparison between measured and calculated (Eq. (2)) incident air kerma for the reference Hologic Selenia Dimensions (device A)

Phantom thickness (cm)	Tube load (mAs)	Tube voltage (kVp)	Measured air kerma (mGy)	Calculated air kerma (mGy)	Relative difference (%)
2.0	37.5	26	2.11 ± 0.08	2.06 ± 0.15	-2.6
2.5	40	27	2.61 ± 0.10	2.54 ± 0.19	-2.8
3.0	37.5	28	2.74 ± 0.11	2.71 ± 0.20	-1.3
3.5	40	29	3.33 ± 0.13	3.24 ± 0.24	-2.7
4.0	50	29	4.14 ± 0.17	4.12 ± 0.31	-0.4
4.5	50	30	4.66 ± 0.19	4.59 ± 0.34	-1.6
5.0	55	31	5.80 ± 0.23	5.60 ± 0.42	-3.6
5.5	60	32	7.08 ± 0.28	6.73 ± 0.50	-5.3
6.0	65	33	8.18 ± 0.33	7.99 ± 0.59	-2.4
6.5	70	34	9.78 ± 0.39	9.40 ± 0.70	-4.1
7.0	80	35	12.03 ± 0.48	11.69 ± 0.87	-2.9
7.5	80	36	13.02 ± 0.52	12.68 ± 0.94	-2.7
8.5	80	38	15.19 ± 0.61	14.78 ± 1.10	-2.8
8.5	90	42	21.52 ± 0.86	20.55 ± 1.53	-4.7

Data for measured air kerma and calculated air kerma are presented as mean ± standard deviation. Note that the exposure parameters were chosen as close as possible to the corresponding automatic exposure control settings for a given phantom thickness

**Table 2:** Values of the coefficients of Eq. (2), (3), and (5) evaluated for the reference Hologic Selenia Dimensions (device A)

Parameter	Y_0_ at 32 kVp (mGy/mAs)	α (mGy/(kVp^2^ ·mAs))	β (mGy/(kVp·mAs))	γ (mGy/mAs)	a (kVp/cm)	b (a.u.)
Value	0.094 ± 0.005	(5.70 ± 0.86)·10^−5^	(3.77 ± 0.56)·10^−3^	(− 8.44 ± 0.89)·10^−2^	20.32 ± 1.97	− 1.04 ± 0.03

Data are presented as mean ± standard deviation. These values were employed to calculate the incident air kerma and 2ABD. Notice that these values depend on the anode-filter combination (W-Al in our case)

### Evaluation of *m*

**Fig. 1:**
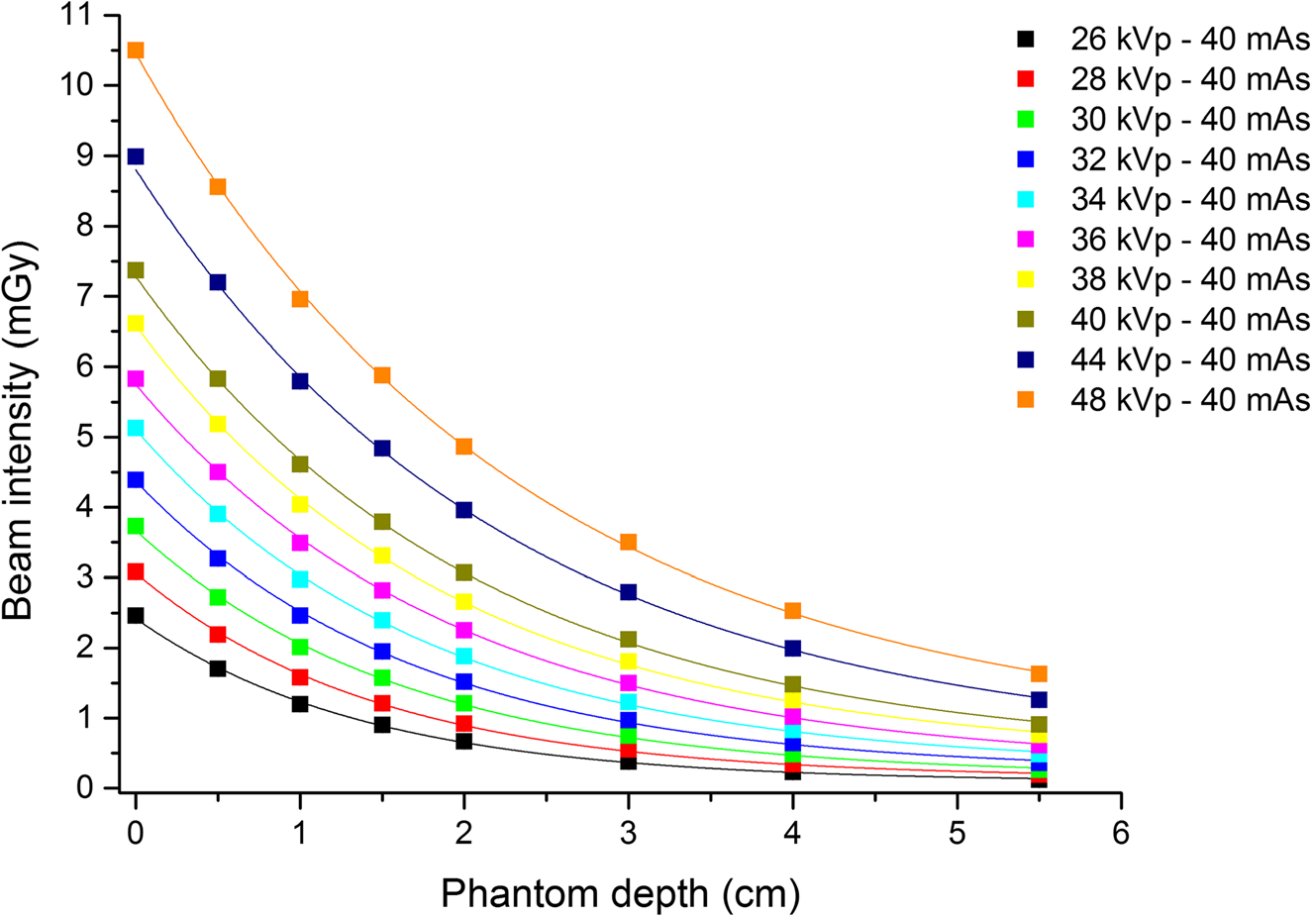
Beam intensity as a function of the phantom depth for different tube voltage values (26–48 kVp). An exponential relationship (Eq. (4)) was employed to fit the data and obtain the *m* values for different tube voltages

**Fig. 2:**
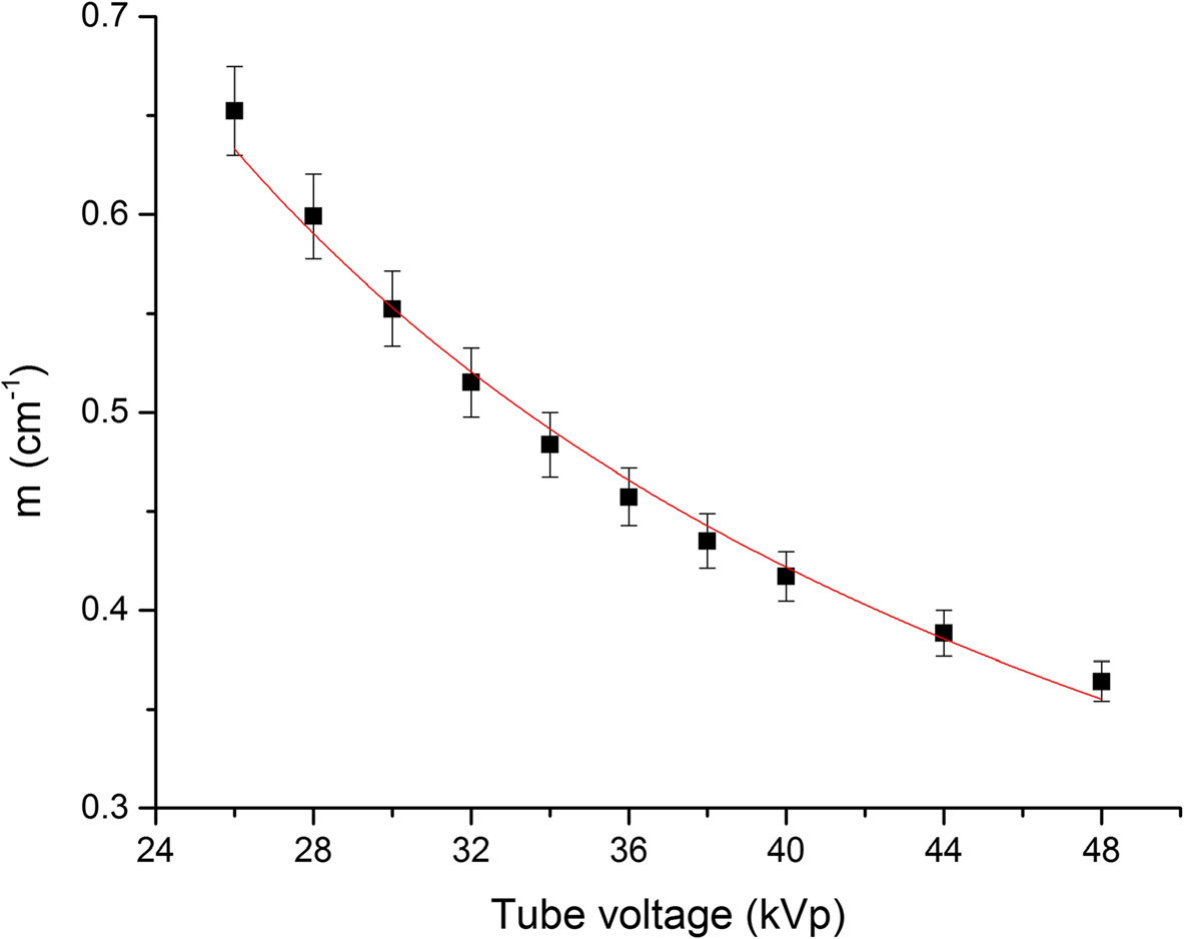
Coefficient *m* estimated from measurements in the phantom, expressed as a function of tube voltage. A power function (Eq. (5)) was applied to model the *m* as a function of the tube voltage values

Equation (4) was employed to fit the beam intensity as a function of the phantom depth (Fig. 1). The *m* values obtained for different tube voltages (from 26 to 48 kVp) are presented in Table 3 and graphically in Fig. 2. The parameters of the fit (*a* and *b*) are reported in Table 2.

**Table 3:** Estimation of the *m* values for different tube voltage values

kVp	m (cm^-1^)
26	0.65 ± 0.03
28	0.60 ± 0.02
30	0.55 ± 0.02
32	0.52 ± 0.02
34	0.48 ± 0.02
36	0.46 ± 0.01
38	0.44 ± 0.01
40	0.42 ± 0.01
44	0.39 ± 0.01
48	0.36 ± 0.01

Data for *m* values are presented as mean ± standard deviation. The *m* values were obtained by fitting experimental data through Eq. (4), as shown in Fig. 1. The fitting parameters of Eq. (4) are presented in Table 2

### Calculation of 2ABD

The 2ABD values computed through Eq. (1) from the exposure parameters were compared with AGD associated to different breast glandularities. A good agreement was found between 2ABD and AGD corresponding to 100% of gland tissue (Table 4). Discrepancies between the two quantities ranged from -6 to 7% by considering all devices. All values were in good agreement within the respective uncertainties.

**Table 4:** Comparison between 2ABD computed from Eq. (1) and the average glandular dose (AGD) calculated by the Dance approach for 100% glandularity

Mammographic device	Phantom thickness (cm)	Tube load (mAs)	Tube voltage (kVp)	2ABD (mGy)	AGD 100% glandularity (mGy)	Relative difference (%)
Hologic-A	3.0	37.5	28	0.94 ± 0.19	1.00 ± 0.20	-6.0
Hologic-A	3.5	40	29	1.05 ± 0.20	1.07 ± 0.21	-1.9
Hologic-A	4.0	50	29	1.21 ± 0.23	1.21 ± 0.24	0.0
Hologic-A	4.5	50	30	1.26 ± 0.23	1.27 ± 0.25	-0.8
Hologic-A	5.0	55	31	1.45 ± 0.26	1.46 ± 0.29	-0.7
Hologic-A	5.5	60	32	1.66 ± 0.30	1.67 ± 0.33	-0.6
Hologic-A	6.0	65	33	1.87 ± 0.33	1.89 ± 0.38	-1.1
Hologic-A	6.5	70	34	2.12 ± 0.37	2.14 ± 0.43	-0.9
Hologic-A	7.0	80	35	2.51 ± 0.44	2.57 ± 0.51	-2.3
Hologic-B	3.5	30	29	1.07 ± 0.20	1.14 ± 0.23	-6.1
Hologic-B	4.5	35	30	1.20 ± 0.22	1.22 ± 0.24	-1.6
Hologic-B	5.5	42.5	32	1.61 ± 0.29	1.64 ± 0.33	-1.8
Hologic-B	6.5	50	34	2.06 ± 0.37	2.09 ± 0.42	-1.4
Fuji-C	3.5	28	29	1.19 ± 0.24	1.21 ± 0.24	-1.7
Fuji-C	4.5	32	32	1.63 ± 0.30	1.56 ± 0.31	4.5
Fuji-C	5.5	42	33	2.06 ± 0.37	2.04 ± 0.41	1.0
Fuji-C	6.5	50	35	2.66 ± 0.47	2.65 ± 0.53	0.4
Fuji-C	4.5^*^	45	32	2.30 ± 0.43	2.15 ± 0.43	7.0
Fuji-C	6.5^*^	63	35	3.35 ± 0.59	3.27 ± 0.65	2.4
Fuji-D	3.5	28	30	1.30 ± 0.26	1.38 ± 0.28	-5.8
Fuji-D	4.5	32	32	1.59 ± 0.30	1.60 ± 0.32	-0.6
Fuji-D	5.5	50	33	2.40 ± 0.43	2.43 ± 0.49	-1.2
Fuji-D	6.5	56	35	2.91 ± 0.50	2.87 ± 0.57	1.4
Fuji-D	3.5^*^	32	30	1.49 ± 0.28	1.55 ± 0.31	-3.9
Fuji-D	4.5^*^	45	32	2.24 ± 0.43	2.20 ± 0.44	1.8
Fuji-D	5.5^*^	63	34	3.35 ± 0.61	3.22 ± 0.64	4.0
Fuji-D	6.5^*^	71	35	3.69 ± 0.65	3.56 ± 0.71	3.7

Data for 2ABD and AGD 100% glandularity are presented as mean ± standard deviation. The comparison was carried out by considering all mammographic devices. The exposure parameters were chosen as close as possible to the corresponding automatic exposure control settings for a given phantom thickness. For the AGD calculation by the Dance method, the measured air kerma was employed. For the Amulet Innovality devices both the standard acquisition mode and high-resolution acquisition mode (^*^) were employed

### Comparison of 2ABD in DM and DBT

**Fig. 3:**
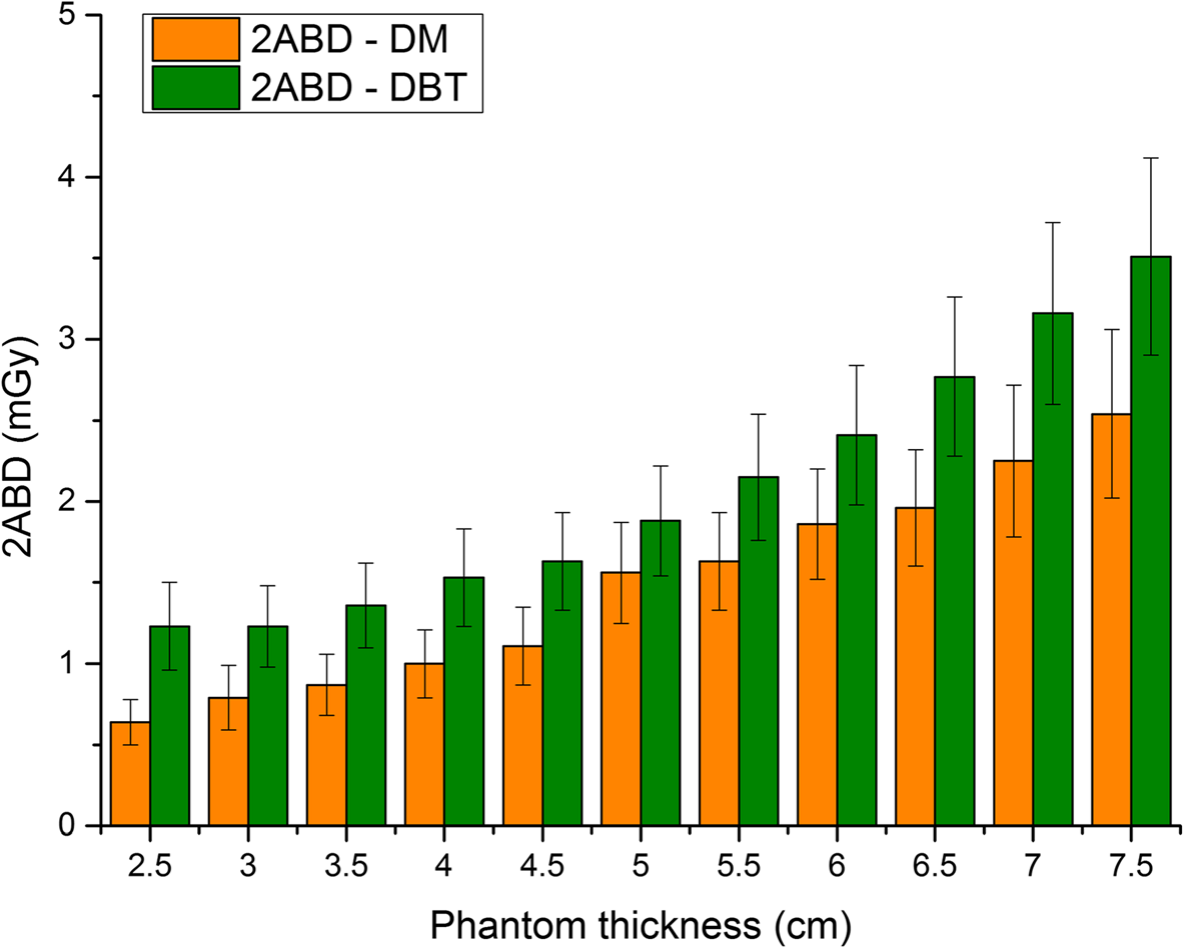
Comparison between the 2ABD computed for digital mammography (DM) and digital breast tomosynthesis (DBT) for the same phantom thickness. The exposure parameters and the complete set of data are presented in Table 5. DM values are referred to a single view procedure

The comparison between the 2ABD calculated for DBT and that calculated for DM for the same phantom thicknesses on device A is shown in Fig. 3. A single view DM was considered in this comparison. The exposure parameters (selected by the AEC system) and the complete set of 2ABD and AGD data are presented in Table 5.

**Table 5:** Comparison between 2ABD in digital mammography and digital breast tomosynthesis for different phantom thicknesses on the reference Hologic Selenia Dimensions (device A)

Phantom thickness (cm)	Digital mammography (single view) (W/Rh and W/Ag^*^)			Digital breast tomosynthesis (W/Al)		
	Tube load (mAs)	Tube voltage (kVp)	2ABD (mGy)	Tube load (mAs)	Tube voltage (kVp)	2ABD (mGy)
2.5	50	26	0.49 ± 0.11	39	27	0.95 ± 0.21
3.0	67	26	0.61 ± 0.15	38	28	0.95 ± 0.19
3.5	72	27	0.67 ± 0.15	40	29	1.05 ± 0.20
4.0	82	28	0.77 ± 0.16	49	29	1.18 ± 0.23
4.5	98	28	0.85 ± 0.18	50	30	1.26 ± 0.23
5.0	134	29	1.20 ± 0.24	55	31	1.45 ± 0.26
5.5	138	30	1.26 ± 0.23	60	32	1.66 ± 0.30
6.0	155	31	1.43 ± 0.26	65	33	1.86 ± 0.33
6.5	161	32	1.51 ± 0.28	71	34	2.13 ± 0.38
7.0^*^	159	30	1.73 ± 0.36	78	35	2.43 ± 0.43
7.5^*^	174	31	1.96 ± 0.40	82	36	2.70 ± 0.47

Data for 2ABD are presented as mean ± standard deviation. In DM modality, the W-Rh anode-filter combination was selected by the AEC system for a range of 2.5–6.5 cm of phantom thickness, while for a thickness of 7 cm and 7.5 cm the W-Ag (^*^) was automatically set. On the other hand, only the W-Al option was available for DBT

The 2ABD values for DBT acquisitions were higher than those obtained in DM for all phantom thicknesses. The maximum difference was expressed by the smallest phantom thickness (2.5 cm), in whose case the 2ABD for DBT resulted 92% higher than the 2ABD for DM. However, the median difference was found to be 44% with respect to DM values. The minimum difference between the two quantities was referred to the 5-cm thick phantom condition in which the 2ABD in DBT was 21% higher than 2ABD in DM.

## Discussion

In spite of its relatively recent introduction, DBT is widely employed both for diagnostic and screening examinations in order to increase the sensitivity and specificity of DM [15, 16]. However, since the breast gland tissue is highly radiosensitive [17], dosimetry monitoring of DBT procedures is essential to ensure the best cost-benefit compromise of this technique. Given that DBT is also employed for breast cancer screening [18–21], a practical operational dosimetry index should be adopted in order to guarantee a fast and reliable monitoring of the absorbed dose.

The current approach adopted for evaluating the absorbed dose in DBT procedures is based on the calculation of the AGD through the method by Dance et al. [10–12]. This method provides a reliable dosimetry measure related to the ionising radiation risk, but its practical implementation could be problematic. In fact, the AGD cannot be directly measured nor computed if the *k*_*a,i*_ is not measured or estimated [11]. Additionally, this approach is based on correction factors tabulated as a function of the beam quality, patient age, projections angle, and breast thickness; interpolation is almost always required for accurately computing the AGD.

For these reasons, the aim of this work was to present a simple model to individually evaluate the average absorbed dose in DBT procedures. The 2ABD index is proposed as a simple phantom approximation of the average dose absorbed by the breast in DBT examinations. 2ABD can be easily computed through Eq. (1) from exposure and geometric parameters which can be found in the DICOM header of each DBT examination. Specifically, to apply our method for a given anode-filter combination, only the knowledge of the tube voltage, the tube load, the breast thickness, the FSD, and the x-ray tube yield is required. Notably, the x-ray tube yield is usually assessed (even at different tube voltages) in periodic quality controls of mammographic devices [9].

The proposed method is firstly based on the estimation of the *k*_*a,i*_ on the breast surface through Eq. (2). The air kerma calculation model was well verified by experimental measurements that confirmed a linear relationship with tube load and a polynomial relationship with tube voltage. More in detail, the comparison between calculated and measured *k*_*a,i*_ under a number of conditions close to clinical settings showed a good agreement within the uncertainties (Table 1); the mean difference between the two quantities over all conditions was around 3%. Even though the computed *k*_*a,i*_ resulted slightly lower with respect to the measured one in every condition (*i.e.*, a little bias was expressed), the maximum differences between the two quantities was only 5.3% which appears to be acceptable for our purpose to give an estimate of the average absorbed dose in a phantom approximating the breast.

The *k*_*a,i*_ calculation model for DBT could be of practical interest in many situations (*e.g.*, fast evaluation of x-ray tube output). This method could be used in different conditions or devices with reasonable accuracy and relative simplicity. Indeed, except for the x-ray tube yield at 32 kVp which should be evaluated in order to correctly apply Eq. (2), no other measurements must be performed to calculate the *k*_*a,i*_: since all the required parameters are provided in Table 2, it should be sufficient to apply Eq. (2) with *Y*_tb_ (32 kVp) evaluated for the considered mammographic device. Of note, by using this approach, the *η* factor in Eq. (2) is assumed to be independent from the tube voltage. However, since *α, β*, γ, and *Y*_0_ (32 kVp) are provided, this approximation allows to calculate the *k*_*a,i*_ for all the possible mammographic devices by only making a single measurement of *Y*_tb_ at 32 kVp.

In order to estimate 2ABD, the coefficient *m* was evaluated for a wide range of tube voltages (Fig. 1) and modelled through Eq. (5) (Fig. 2 and Table 3). An approximation was made by using the exponential decay model in order to obtain *m* (the polychromatic nature of the x-ray beam was neglected, considering the beams quasi-monochromatic). However, experimental data fit quite well the exponential model, which appears to be acceptable (Fig. 1). In particular, the experimental data appear to be reasonably compatible with the proposed model proposed in Eq. (5), especially within the 30–40 kVp energy range.

The 2ABD was compared to AGD for different breast glandularities. Our results showed that 2ABD matches the AGD values related to 100% breast gland tissue (Table 4). Notice that several exposure conditions and different mammographic devices were considered in our study. More in detail, the discrepancies between the two quantities ranged from -6 to 7%, by considering all the conditions. These results emphasise the high flexibility and accuracy of the method. It should be noted that the comparison carried out on the Amulet Innovality devices took into account both the standard acquisition mode (± 7.5° angular range) and the high-resolution acquisition mode (± 20° angular range). Nevertheless, the higher angular range seemed to have negligible influence on 2ABD accuracy. In addition, in order to take into account different glandularities, a rough method based on the mass density of glandular and adipose tissue was presented Appendix. The method permits to estimate the AGD with an accuracy ranging from ~ 7 (when the glandularity is 100%) to ~ 23% (when the glandularity is 20%), by only knowing the breast glandularity and the 2ABD value. Examples of comparison between the AGD computed from the Dance approach and AGD estimated from the 2ABD are reported in Table 6.

**Table 6:** Comparison between AGD computed from 2ABD and the AGD calculated by the Dance approach for different glandularities

Glandularity (%)	Phantom thickness (cm)	Tube load (mAs)	Tube voltage (kVp)	AGD from 2ABD (mGy)	AGD by Dance (mGy)	Relative difference (%)
100	3	37.5	28	0.94 ± 0.19	1.00 ± 0.20	-6.0
100	4	50	29	1.21 ± 0.23	1.21 ± 0.24	0.0
100	5	55	31	1.45 ± 0.26	1.46 ± 0.29	-0.7
100	6	65	33	1.87 ± 0.33	1.89 ± 0.38	-1.1
100	7	80	35	2.57 ± 0.44	2.57 ± 0.31	-2.3
80	3	37.5	28	0.96 ± 0.19	1.06 ± 0.21	-9.4
80	4	50	29	1.24 ± 0.24	1.30 ± 0.26	-4.6
80	5	55	31	1.48 ± 0.27	1.58 ± 0.32	-6.3
80	6	65	33	1.91 ± 0.34	2.04 ± 0.41	-6.4
80	7	80	35	2.56 ± 0.45	2.78 ± 0.56	-7.9
60	3	37.5	28	0.98 ± 0.20	1.12 ± 0.22	-12.5
60	4	50	29	1.26 ± 0.24	1.40 ± 0.28	-10.0
60	5	55	31	1.51 ± 0.27	1.70 ± 0.34	-11.2
60	6	65	33	1.95 ± 0.34	2.21 ± 0.44	-11.8
60	7	80	35	2.62 ± 0.46	3.01 ± 0.60	-13.0
40	3	37.5	28	1.00 ± 0.20	1.19 ± 0.24	-16.0
40	4	50	29	1.29 ± 0.25	1.50 ± 0.30	-14.0
40	5	55	31	1.55 ± 0.28	1.84 ± 0.37	-15.8
40	6	65	33	2.00 ± 0.35	2.41 ± 0.48	-17.0
40	7	80	35	2.68 ± 0.47	3.29 ± 0.66	-18.5
20	3	37.5	28	1.03 ± 0.21	1.26 ± 0.25	-18.3
20	4	50	29	1.32 ± 0.25	1.61 ± 0.32	-18.0
20	5	55	31	1.59 ± 0.28	2.00 ± 0.40	-20.5
20	6	65	33	2.04 ± 0.36	2.62 ± 0.52	-22.1
20	7	80	35	2.74 ± 0.48	3.58 ± 0.72	-23.5

Data for AGD from 2ABD and for AGD by Dance are presented as mean ± standard deviation. The comparison was carried out by considering the reference mammographic devices (Hologic, device A). The exposure parameters were chosen as close as possible to the corresponding AEC settings for a given phantom thickness. For the AGD by Dance, the measured air kerma was employed

To further investigate the dosimetry properties of DBT imaging, a comparison between 2ABD calculated for DM and DBT modalities was performed on device A for the same phantom thicknesses (Table 5 and Fig. 3). As expected, 2ABD values in DBT resulted higher 2ABD with respect to 2ABD calculated in DM for each phantom thickness. This aspect is clearly related to differences in exposure parameters as well as to differences in x-ray spectra between the two modalities (Table 5). These results confirm that DBT expresses higher absorbed dose than a single view DM up to 92% for the same phantom (2.5-cm thickness). Given the employed AEC conditions, it is reasonable to expect similar results even in clinical examinations.

Note that in this work only two DBT device models were employed, and two different tube angular ranges were explored. Even though in the abovementioned conditions the proposed method can be applied with relatively acceptable accuracy, additional investigations are required for different DBT devices, especially for wider angular tube ranges, on the homogeneity of dose distribution and dosimeter angular dependence. This simple model should be revised accordingly.

It is important to recognise that our absorbed dose evaluation model is entirely developed in a homogeneous phantom, therefore 2ABD represents the mean absorbed dose in an homogeneous phantom which approximates a 100% glandularity. Thus, for a theoretical 100% glandular breast, the accuracy achieved in AGD estimation is maximum (2ABD approximates AGD within ~ 7%). However, it should be emphasised that the discrepancies between 2ABD and AGD increase with decreasing breast glandularity. Additionally, it should be remarked that 2ABD could be considered only as surrogate of the average absorbed dose in a real breast, which is usually characterised by different tissues and dose distribution. Furthermore, a homogeneous object is not properly suitable to accurately simulate the breast. Therefore, heterogeneous (*i.e.*, with different percentages of glandular/adipose tissue composition) and breast-shaped phantoms could be useful to test this method in further studies.

In conclusion, despite the highly simplified approach, the presented method allowed to calculate the mean absorbed dose in a phantom with reasonable accuracy by only knowing the tube voltage, tube load, breast thickness, FSD, and the x-ray tube yield. Values of Tables 2 and 3 can be employed for calculating 2ABD; the *k*_*a,i*_ can be computed accurately for at least two different mammographic devices.

## Data Availability

All data generated or analysed during this study are included in this published article.

## Appendix

### Calculation of incident air kerma and X-ray tube yield model

Equation (2) describes an approximation in which the *η* factor is independent from the tube voltage. This assumption simplifies the calculation of the incident air kerma for different mammographic devices since only one measurement of *Y*_tb_ (at 32 kVp) is required. On the other hand, this method results in accuracy loss < 10% in the energy range of interest.

In order to estimate the air kerma, a more correct and accurate approach requires a set of measurements of *Y*_tb_ (kVp) by the user and Eq. (2) then could be simplified in:

6 $$ {k}_{a,i}={Y}_{tb}\left(\mathrm{kVp}\right)\cdot \mathrm{mAs}\cdot {\left(\frac{\mathrm{FSD}}{\mathrm{FSD}\hbox{-} \mathrm{T}}\right)}^2 $$

Furthermore, in a general case, the x-ray tube yield (*Y*) at a fixed source distance could be modelled as function of the tube voltage by the following relationship:

7 $$ Y\left(\mathrm{kVp}\right)=\left({c}_1\times {\mathrm{kVp}}^2+{c}_2\cdot \mathrm{kVp}+{c}_3\right) $$

in which c_i_ are fitting parameters.

Notice that, since device A was employed as reference in this study, it results always *η* = 1 for this case. Therefore, the term (α·kVp^2^ + β·kVp + γ) is just *Y*_0_ (kVp), as suggested by Eq. (7), and the form of Eq. (2) is the same of Eq. (6).

### Estimation of AGD from 2ABD

In order to estimate the AGD from 2ABD for a general breast glandularity, the following approximations are needed: (1) our phantom is considered equivalent to a 100% glandular composition, (2) the energy absorbed by glandular tissue is considered linearly related to the breast glandularity.

From approximation (1) it follows that *ρ*_ph_ ≈ *ρ*_gl_, where *ρ*_ph_ and *ρ*_gl_ are the density of our phantom and the density of the glandular tissue respectively (${\rho}_{\mathrm{g}\mathrm{l}}\approx 1.04\ \frac{\mathrm{g}}{{\mathrm{cm}}^3}$ [22]). The AGD related to a 100% glandular composition can be written as follows:

8 $$ AGD\left(100\%\right)=\frac{E_{\mathrm{gl}}}{m_{\mathrm{gl}}}\approx 2\mathrm{ABD} $$

where *E*_gl_ and *m*_gl_ are the absorbed energy and the mass of the glandular tissue.

For a phantom/breast with a glandularity *g*, the total mass *m*_tot_(*g*) can be obtained by the following equation:

9 $$ {m}_{\mathrm{tot}}(g)={V}_{\mathrm{tot}}\cdot \left(g\cdot {\rho}_{gl}+\left(1-g\right)\cdot {\rho}_{\mathrm{ad}}\right) $$

where *V*_tot_is the total volume of the breast/phantom.

The corresponding glandular mass in a phantom/breast with glandularity *g* is given by:

10 $$ {m}_g(g)={m}_{\mathrm{tot}}(g)\cdot g={V}_{\mathrm{tot}}\cdot \left(g\cdot {\rho}_{gl}+\left(1-g\right)\cdot {\rho}_{\mathrm{ad}}\right)\cdot g $$

where *ρ*_ad_ is the density of the adipose tissue (${\rho}_{\mathrm{ad}}\approx 0.93\ \frac{\mathrm{g}}{{\mathrm{cm}}^3}$ [22]).

From (2), the energy absorbed by a breast/phantom with a glandularity *g* can be written:

11 $$ {E}_g(g)\approx {E}_g\cdot g $$

Therefore, the AGD related to a breast/phantom with a glandularity *g* is given by the following relationship:

12 $$ AGD(g)=\frac{E_g(g)}{m_g(g)}\approx \frac{2 ABD\cdot {\rho}_{\mathrm{gl}}}{g\cdot {\rho}_{\mathrm{gl}}+\left(1-g\right)\cdot {\rho}_{\mathrm{ad}}} $$

## Abbreviations

2ABD: Average absorbed breast dose
AEC: Automatic exposure control
AGD: Average glandular dose
DBT: Digital breast tomosynthesis
DM: Digital mammography
FSD: Focus-to-support distance
*k*_*a,i*_: Incident air kerma
